# SHED-Secretome Gel Promotes Early Alveolar Bone Healing via Osteoblast-Osteoclast Modulation

**DOI:** 10.1590/0103-644020256808

**Published:** 2025-12-08

**Authors:** Nikmatus Sa’adah, Indeswati Diyatri, Mohammed Ahmed Aljunaid, Agus Aan Adriansyah, Huda Rashad Qaid, Raed Labib, Rini Devijanti Ridwan

**Affiliations:** 1 Doctoral Study Program, Faculty of Dental Medicine, Universitas Airlangga, Surabaya, Indonesia; 2 Department of Biomedic, Faculty of Dental Medicine, Institut Ilmu Kesehatan Bhakti Wiyata, Kediri, Indonesia; 3 Research Center, Faculty of Dental Medicine, Universitas Airlangga, Surabaya, Indonesia; 4 Department of Oral Biology, Faculty of Dental Medicine, Universitas Airlangga, Surabaya, Indonesia; 5 Department of Oral and Dental Medicine, Faculty of Medicine, Taiz University, Taiz, Yemen; 6 Department of Public Health, Faculty of Health, Universitas Nahdlatul Ulama Surabaya, Surabaya, Indonesia; 7 Faculty of Oral and Dental Medicine, Al-Saeed University, Taiz, Yemen; 8 Department of Oral Surgery, Faculty of Dental Medicine, 21 September University of Medical and Applied Sciences, Sana’a, Yemen

**Keywords:** stem cell-derived secretome, dental pulp stem cells, osteoblasts, osteoclasts, tooth extraction

## Abstract

Post-extraction alveolar bone healing is crucial for dental rehabilitation, depending on the balance between osteoblast and osteoclast activity. This study evaluated the potential of SHED (stem cells from human exfoliated deciduous teeth)-derived secretome gel in promoting bone regeneration. Eighteen male *Rattus norvegicus* were divided into three groups: negative control (placebo gel), positive control (absorbable gelatin sponge), and treatment (SHED-secretome gel). Maxillary first molars were extracted, and the respective materials were applied to the sockets. On day 7, histological samples were analyzed. Results showed that the SHED-secretome group exhibited significantly higher osteoblast and osteoclast numbers compared to controls, indicating active and balanced bone remodeling. The bone matrix appeared more organized, suggesting accelerated early healing. Statistical analysis (Kruskal-Wallis and Mann-Whitney U with Bonferroni correction) confirmed significant differences (p < 0.01) in osteoblast and osteoclast counts across groups. In conclusion, SHED-secretome gel enhances early alveolar bone healing by promoting coordinated osteoblast-osteoclast activity essential for physiological bone remodeling.

## Resumo

A cicatrização do osso alveolar pós-extração é crucial para a reabilitação dentária, dependendo do equilíbrio entre a atividade dos osteoblastos e dos osteoclastos. Este estudo avaliou o potencial do gel secretoma derivado de SHED (células-tronco de dentes decíduos esfoliados humanos) na promoção da regeneração óssea. Dezoito Rattus norvegicus machos foram divididos em três grupos: controle negativo (gel placebo), controle positivo (esponja de gelatina absorvível) e tratamento (gel de secretoma SHED). Os primeiros molares maxilares foram extraídos e os respectivos materiais aplicados nas cavidades. No sétimo dia, amostras histológicas foram analisadas. Os resultados mostraram que o grupo SHED-secretoma apresentou números significativamente mais elevados de osteoblastos e osteoclastos em comparação com os controles, indicando uma remodelação óssea ativa e equilibrada. A matriz óssea parecia mais organizada, sugerindo uma cicatrização precoce acelerada. A análise estatística (Kruskal-Wallis e Mann-Whitney U com correção de Bonferroni) confirmou diferenças significativas (p < 0,01) nas contagens de osteoblastos e osteoclastos entre os grupos. Em conclusão, o gel de secretoma SHED melhora a cicatrização precoce do osso alveolar, promovendo a atividade coordenada de osteoblastos e osteoclastos, essencial para a remodelação óssea fisiológica.

**Figure:**
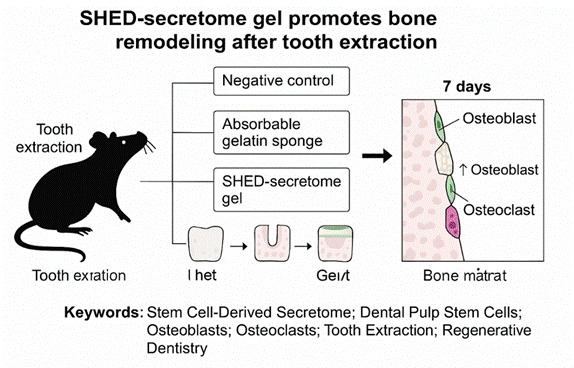

## Introduction

Tooth extraction triggers a sequence of inflammatory, proliferative, and bone remodeling phases. Initially, clot formation and immune cell infiltration stabilize the wound, followed by proliferation of fibroblasts, endothelial cells, and osteoprogenitor cells to restore tissue integrity. Effective healing is crucial for maintaining alveolar ridge structure to support implants or prostheses. However, rapid horizontal and vertical bone loss often occurs within weeks after extraction. This is driven by an imbalance where osteoclastic resorption exceeds osteoblastic formation during early healing^1^ ^,^ ^2^. Thus, strategies to promote faster and more balanced bone regeneration are urgently needed in clinical dentistry.

Osteoblasts are specialized mesenchymal-derived cells responsible for synthesizing and mineralizing the extracellular bone matrix through the secretion of type I collagen and osteogenic proteins such as osteocalcin and alkaline phosphatase. In contrast, osteoclasts are multinucleated cells of hematopoietic origin that degrade bone tissue by secreting hydrochloric acid and proteolytic enzymes, a process essential for bone turnover and structural adaptation. Under physiological conditions, the activities of osteoblasts and osteoclasts are tightly coupled through complex signaling pathways involving receptor activator of nuclear factor κB ligand (RANKL), osteoprotegerin (OPG), and various cytokines, thereby ensuring balanced bone remodeling^3^ ^,^ ^4^. When osteoclast activity dominates, the socket undergoes rapid bone loss and delayed healing. Therefore, promoting osteoblastogenesis and inhibiting excessive osteoclastic resorption are key goals in enhancing post-extraction bone regeneration.

Stem cell-based regenerative therapies have shifted focus from cellular engraftment to the paracrine effects of secreted factors. The stem cell secretome, comprising cytokines, growth factors, and extracellular vesicles, can modulate inflammation, stimulate angiogenesis, and enhance osteogenesis^5^. Compared to direct stem cell transplantation, secretome-based approaches avoid risks such as immune rejection, tumorigenicity, and poor cell survival, while offering low immunogenicity, long-term storage, and minimally invasive delivery. In craniofacial bone regeneration, these attributes enable standardized protocols and maintain regenerative efficacy through paracrine signaling^6^ ^,^ ^7^. These attributes make secretome gel an appealing alternative for bone tissue engineering, particularly in dental applications.

Stem cells from human exfoliated deciduous teeth (SHED) are mesenchymal stem cells with high proliferative and osteoinductive potential. SHED-derived secretome contains pro-regenerative factors that promote osteoblast differentiation and inhibit osteoclast formation^8^. Delivering SHED secretome in gel form enables localized, sustained release of bioactive components at the extraction site, with the hydrogel serving as a scaffold and protective reservoir to preserve activity over time. This controlled release supports osteoblast proliferation, angiogenesis, and inflammation modulation during early socket healing, while its injectable and moldable nature ensures optimal socket adaptation. As a minimally invasive, patient-friendly approach, this strategy aligns with current regenerative dental biomaterial trends, offering high efficacy with reduced complexity and strong potential for clinical translation in oral and maxillofacial bone repair^9^ ^,^ ^10^.

The rat model (*Rattus norvegicus*) is widely used for post-extraction healing studies due to its bone turnover characteristics comparable to humans. Day 7 post-extraction represents a critical transition from inflammation to bone formation, characterized by active osteoblast and osteoclast involvement. Histological quantification of these cells serves as a reliable indicator of regenerative potential and biomaterial effects. This study utilizes that window to assess the impact of SHED-secretome gel on socket healing. Focusing on the early proliferative phase, the research evaluates this cell-free strategy’s role in modulating alveolar socket cellular activity^11^. The findings aim to provide mechanistic evidence supporting SHED-derived products in accelerating and optimizing healing outcomes

This study aims to investigate the effects of SHED-derived secretome gel on osteoblast and osteoclast populations in alveolar sockets of *Rattus norvegicus* at day 7 post-tooth extraction. We hypothesize that secretome application accelerates bone healing by enhancing osteoblast proliferation and reducing osteoclastic resorption. Findings from this study may contribute to the development of sustainable, biologically active therapeutic approaches in dental regenerative medicine and socket preservation techniques.

## Materials and methods

### Study Design

This research has received approval from Universitas Airlangga Faculty of Dental Medicine Health Research Ethical Clearance Commission Number 0007/HRECC.FODM/I/2025. This research was conducted at the Biochemistry Laboratory of the Faculty of Medicine, Airlangga University, and the Research Center of the Faculty of Dental Medicine, Airlangga University. This study was designed as an *in vivo* experimental laboratory study with a post-test control group design. This research analyzed the number of osteoblasts and osteoclasts in the wound healing process after tooth extraction, after administration of SHED-derived secretome gel, and evaluated their contribution in supporting the wound healing process after tooth extraction.

### Preparation of SHED-Derived Secretome Gel

SHED metabolites are purified from the SHED provided by the Research Centre, Faculty of Dental Medicine, Universitas Airlangga. The SHED was cultured from passages 4 in Dulbecco’s modified Eagle medium. SHED culture medium was purified using the dialysis method to remove waste products of metabolism that were not useful, resulting in beneficial results of metabolites that contained several cytokines, growth factors, and exosomes. At passage 4, SHED-conditioned medium (secretome) was collected after 48 hours of serum-free incubation. The supernatant was centrifuged at 3000 rpm for 10 minutes, filtered through a 0.22 μm filter, and stored at −80°C. The secretome was then incorporated into hydroxypropyl methylcellulose (HPMC) gel as the carrier matrix for local delivery, forming the SHED-secretome gel.

### Animal Grouping and Tooth Extraction Procedure

The animals were randomly assigned into three groups (n = 6 per group): the negative control group, which received a placebo gel, the positive control group, which received absorbable gelatin sponge, and the treatment group, which received SHED-secretome gel. All animals were anesthetized with ketamine (40 mg/kg) injected intramuscularly. Left mandibular incisors were extracted under sterile conditions using a small dental elevator adapted for rodents. Post-extraction sockets were immediately filled with 1 mL of gel each using a micropipette. Animals were monitored postoperatively for any signs of infection or distress.

### Tissue Collection and Histological Processing

On day 7 post-extraction, animals were euthanized using an overdose of sodium pentobarbital. Mandibular bone segments containing the extraction sockets were harvested, fixed in 10% neutral buffered formalin for 48 hours, decalcified in 10% EDTA for 2 weeks, and embedded in paraffin. Serial sagittal sections (5 μm thickness) were cut and stained with hematoxylin and eosin (HE) for histological examination.

### Histological Evaluation and Cell Counting

Histological sections were examined under a light microscope at 400× magnification. Osteoblasts were identified as mononuclear cuboidal cells lining the newly formed bone surface, while osteoclasts were recognized as large multinucleated cells adjacent to bone-resorbing areas. The number of osteoblasts and osteoclasts was counted manually in 8 fields of view using a light microscope. The mean value per field was calculated for each sample and used for statistical analysis.

### Statistical Analysis

Statistical analysis was performed using SPSS. Data normality was assessed using the Shapiro-Wilk test, and the results indicated that the data were not normally distributed. Therefore, differences in the number of osteoblasts and osteoclasts among the three groups (negative control, positive control, and SHED-secretome treatment) were analyzed using the Kruskal-Wallis test. This non-parametric approach was selected to accommodate small sample sizes and the observed violation of normality assumptions in biological count data.

## Results

Histological evaluation of the alveolar socket tissues on day 7 post-extraction revealed distinct cellular responses among the experimental groups. In the negative control group, osteoblasts appeared sparsely aligned along the bone surface, with a minimal presence of newly formed woven bone. The surrounding connective tissue exhibited loose fibrous structures with few active bone-forming cells. The positive control group demonstrated moderate osteoblast distribution along trabecular surfaces, while the SHED-secretome gel-treated group exhibited the most pronounced histological activity. A dense alignment of osteoblasts was observed bordering the newly deposited bone matrix, accompanied by increased osteoclast presence adjacent to remodeling areas. These findings suggest that SHED-secretome gel induces active bone turnover with concurrent osteoblast and osteoclast involvement, supporting a remodeling phase of early bone regeneration. The bone surface appeared smoother and more mineralized, with increased cellular organization and evidence of early woven bone formation.

**Figure 1:**
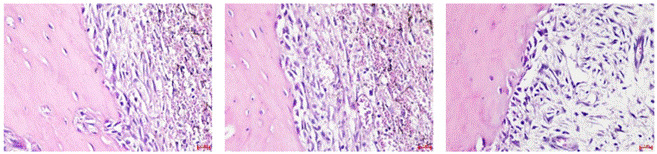
Histological section showing osteoblasts lining the newly formed bones to the alveolus at 400x magnification (left to right: the negative control group, the positive control group, and the treatment group).

Descriptive analysis was conducted to evaluate the number of osteoclasts and osteoblasts observed on day 7 post-extraction across three experimental groups: the negative control group (K7), which received a placebo gel, the positive control group (K7+), which received absorbable gelatin sponge, and the treatment group (K7P), which received SHED-secretome gel. The mean number of osteoclasts was lowest in the K7-group (4.83 ± 0.41), increased in the K7+ group (6.67 ± 0.52), and highest in the K7P group (8.67 ± 0.52). In contrast, the mean number of osteoblasts was also lowest in the K7-group (2.33 ± 0.52), increased in the K7+ group (3.83 ± 0.41), and highest in the K7P group (7.33 ± 0.82). These data suggest that SHED-secretome gel may promote osteoblast proliferation while increasing osteoclastic activity at this stage of healing. 

**Figure 2:**
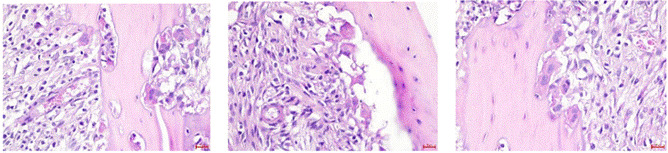
Histological section showing multinucleated osteoclasts adjacent to the bone surface within the alveolar socket at 400× magnification (left to right: the negative control group, the positive control group, and the treatment group).

**Table 1:** Descriptive Data

Parameters	Group	Mean	Min	Max	SD	CI 95%
Osteoclast	K7+	6,67	6	7	0,516	6,12 - 7,21
	K7-	4,83	4	5	0,408	4,40 - 5,26
	K7P	8,67	8	9	0,516	8,12 - 9,21
Osteoblast	K7+	3,83	3	4	0,408	3,40 - 4,26
	K7-	2,33	2	3	0,516	1,79 - 2,88
	K7P	7,33	6	8	0,816	6,48 - 8,19

**Table 2:** Kruskal-Wallis Test

Parameters	Group	Sig. Kruskal-Wallis
Osteoclast	K7+	0,001
	K7-	
	K7P	
Osteoblast	K7+	0,001
	K7-	
	K7P	

**Table 3:** Mann-Whitney Test

Parameters	Group Comparison	Sig. Mann-Whitney	Annotation
Osteoclast	K7+ vs K7-	0,002	There is a significant difference
	K7+ vs K7P	0,003	There is a significant difference
	K7- vs K7P	0,002	There is a significant difference
Osteoblast	K7+ vs K7-	0,004	There is a significant difference
	K7+ vs K7P	0,003	There is a significant difference
	K7- vs K7P	0,003	There is a significant difference

Due to the non-normal distribution of the data, the Kruskal-Wallis test was applied. The results indicated statistically significant differences among the three groups for both parameters. These findings suggest that the type of treatment influenced both osteoclast and osteoblast numbers. Pairwise comparisons using the Mann-Whitney U test further confirmed significant differences. These results indicate that SHED-secretome gel significantly increased both osteoblast and osteoclast activity compared to the control groups, suggesting an active remodeling phase in the healing socket.

## Discussion

The present study demonstrated that the topical application of SHED-secretome gel significantly enhanced alveolar socket healing on day 7 post-extraction, as evidenced by increased osteoblast and osteoclast counts. This concurrent increase indicates that SHED-secretome gel promotes an active remodeling process rather than a simple inhibition of bone resorption^12^ ^,^ ^8^. SHED secretome contains a bioactive mixture of growth factors, cytokines, chemokines, and extracellular vesicles, including BMPs, TGF-β, VEGF, IL-10, and TSG-6, which collectively stimulate osteoblast activity, angiogenesis, and inflammation modulation^5^ ^,^ ^13^. Additionally, SHED-derived extracellular vesicles carry regulatory microRNAs and proteins that enhance cell proliferation, differentiation, and matrix remodeling, creating a favorable environment for accelerated and high-quality bone regeneration.

The observed increase in osteoblast numbers in the SHED-secretome group supports its role in promoting osteogenic differentiation. SHED secretes high levels of BMP-2, VEGF, and TGF-β, which stimulate osteoblast maturation, matrix deposition, and mesenchymal progenitor recruitment^6^ ^,^ ^14^. Extracellular vesicles within the secretome deliver osteoinductive microRNAs such as miR-21 and miR-26a, enhancing lineage commitment via PI3K/Akt and Wnt/β-catenin pathways^15^ ^,^ ^16^. Together, these growth factors and miRNA-loaded vesicles accelerate the transition from granulation tissue to mineralized bone, improving the rate and quality of post-extraction socket healing.

Meanwhile, the increased osteoclast number observed in this group should be interpreted as a physiological part of the remodeling phase. Osteoclasts are required for the resorption of immature matrix, allowing subsequent replacement with mature, mineralized bone tissue. This may involve anti-inflammatory cytokines such as IL-10 and TSG-6, which inhibit RANKL-induced osteoclast differentiation via NF-κB and MAPK pathway modulation^17^ ^,^ ^18^. This transient elevation suggests that SHED-secretome gel accelerates the remodeling cycle rather than inducing excessive resorption. Thus, the higher counts of both osteoblasts and osteoclasts indicate synchronized cellular activity, reflecting enhanced turnover and balanced regeneration of the alveolar bone.

Post-extraction day 7 represents the early proliferative phase of socket healing, when inflammation subsides and osteoblasts begin producing extracellular matrix and initiating woven bone formation^19^ ^,^ ^20^. Without treatment, elevated osteoclast activity can prolong matrix resorption and delay scaffold stability. SHED-secretome gel helps balance osteoclast and osteoblast activity, thus expediting the shift from granulation tissue to woven bone and creating a favorable microenvironment for remodeling into mature lamellar bone.

These observations are consistent with emerging evidence that stem cell-derived secretomes can orchestrate cellular crosstalk in bone healing by modulating both anabolic and catabolic processes. By supporting osteoblast proliferation and maintaining controlled osteoclast function, SHED-secretome gel contributes to a more stable and mature bone matrix in early healing phases^3^. Unlike cell-based approaches, which face challenges related to cell viability, immune compatibility, and regulatory compliance, secretome-based interventions offer a safer profile, reduced immunogenicity, and simplified storage, transport, and clinical application^21^ ^,^ ^22^. In alveolar bone healing, SHED secretome within a hydrogel enables sustained release, enzymatic protection, and retention in the socket, maintaining osteoinductive factor levels to support osteoblast proliferation and matrix formation. The improved osteoblast count in the treatment group likely reflects both the SHED secretome’s bioactivity and the optimized delivery by the gel, highlighting the synergistic potential of biomaterial-based platforms for oral and maxillofacial bone repair.

Our findings add to growing evidence supporting the regenerative potential of SHED-derived products in craniofacial bone repair. Previous studies have shown that SHED-conditioned medium promotes periodontal ligament regeneration, calvarial defect closure, and fracture healing via pro-osteogenic and angiogenic effects^23^ ^,^ ^24^. These benefits stem from its rich content of growth factors, extracellular vesicles, and immunomodulatory cytokines that stimulate osteogenesis and modulate inflammation. However, few studies have explored its application in post-extraction alveolar sockets, a critical site for ridge preservation^25^. Given the socket’s rapid turnover and susceptibility to resorption, our study highlights the SHED secretome's role in maintaining the balance between bone formation and resorption during early healing^26^. This expands its potential in oral and maxillofacial regenerative applications.

This study has several limitations, including assessment at only one time point (day 7), which may not capture the full course of bone healing. Future research should examine multiple time points and apply molecular assays such as RT-PCR and ELISA to quantify osteogenic and osteoclastic markers. Translating these findings from rodent models to humans will require dose optimization and evaluation of long-term outcomes. Overall, SHED-secretome gel shows potential to enhance early alveolar bone healing by modulating osteoblast-osteoclast balance. These results support its promise as a cell-free regenerative approach for dental and oral surgery. Further studies should investigate its role in socket preservation, guided bone regeneration, and implantology.

## Conclusion

SHED-secretome gel enhances early alveolar bone healing after tooth extraction by stimulating osteoblast proliferation and promoting balanced bone remodeling through physiological osteoclast activation. These effects support its potential as a cell-free therapy to accelerate early bone formation and aid socket preservation. The findings suggest its promise for clinical use, warranting further long-term and molecular investigations.
